# Refractive Index Sensor Based on a Metal–Insulator–Metal Waveguide Coupled with a Symmetric Structure

**DOI:** 10.3390/s17122879

**Published:** 2017-12-11

**Authors:** Shubin Yan, Meng Zhang, Xuefeng Zhao, Yanjun Zhang, Jicheng Wang, Wen Jin

**Affiliations:** 1 Science and Technology on Electronic Test and Measurement Laboratory, North University of China, No. 3 Xueyuan Road, Taiyuan 030051, China; ZM_Bulbasaur@163.com (M.Z.); xf_zhao@st.nuc.edu.cn (X.Z.); zhangyanjun@nuc.edu.cn (Y.Z.); 2 School of Science, Jiangsu Provincial Research Center of Light Industrial Optoelectronic Engineering and Technology, Jiangnan University, Wuxi 214122, China; jcwang@jiangnan.edu.cn; 3 Aerospace Science and Technology Corporation, Beijing Institute of Space Long March Vehicle, Beijing 100036, China; jin.wen@126.com

**Keywords:** surface plasmon polaritons, Fano resonance, finite element method, refractive index sensor

## Abstract

In this study, a new refractive index sensor based on a metal–insulator–metal waveguide coupled with a notched ring resonator and stub is designed. The finite element method is used to study the propagation characteristics of the sensor. According to the calculation results, the transmission spectrum exhibits a typical Fano resonance shape. The phenomenon of Fano resonance is caused by the coupling between the broadband spectrum and narrowband spectrum. In the design, the broadband spectrum signal is generated by the stub, while the narrowband spectrum signal is generated by the notched ring resonator. In addition, the structural parameters of the resonators and the structure filled with media of different refractive indices are varied to study the sensing properties. The maximum achieved sensitivity of the sensor reached 1071.4 nm/RIU. The results reveal potential applications of the coupled system in the field of sensors.

## 1. Introduction

Surface plasmon polaritons (SPPs) are charge density waves [1,2] and are formed by the interference of free electrons and electrons on the surface of a metal film, which propagate along the metal surface, and the electric field amplitude decays exponentially in the vertical interface direction [3,4,5,6,7]. SPPs propagate only on the interface of metals and dielectrics; therefore, it can break through the traditional optical diffraction limit [8]. In addition, the size of the SPP model is small, and nanoscale optical transmission, processing, and control can be realized [9,10,11]. Optical devices based on SPP waveguide structures have been extensively studied, such as in filters [12,13,14], diodes [15,16,17], and optical switches [18,19,20].

In recent years, optical phenomena such as Fano resonance [21,22,23] and plasmon-induced transparency [24,25] caused by metal–insulator–metal (MIM) waveguide coupling cavities, which are sensitive to the surrounding environment, have attracted the interest of domestic and foreign researchers to study SPP sensors [26,27,28]. Thus, many sensors based on the MIM waveguide have been investigated and reported. Zhao et al. [29] reported an asymmetric plasmonic waveguide that consisted of two silver baffles and coupled ring resonators, which has a maximum refractive index sensitivity of 718 nm/RIU. Zhang et al. [21] showed a gear-shaped nanocavity which was on the basis of the MIM waveguide that has a maximum refractive index sensitivity of 744 nm/RIU. Zhang et al. [30] showed a plasmonic nanosensor that consisted of coupled double rectangular resonators, which has a refractive index sensitivity of 596 nm/RIU. However, these plasmonic sensors currently exhibit low sensitivity, which is an enormous challenge to researchers. 

In this study, a structure consisting of MIM waveguides coupled with the stub and notched ring resonator was applied as a plasmonic refractive index nanosensor. The structure was distributed by the transmission spectra and magnetic *H_z_* field with a perfectly matched layer absorbing boundary condition and was calculated by the finite element method. We varied the coupling distance between the stub and notched ring resonator, and the external diameter of the notched ring resonator and length of the stub to study its sensing characteristics and refractive index sensitivity.

## 2. Model and Analytical Method

**Figure 1:**
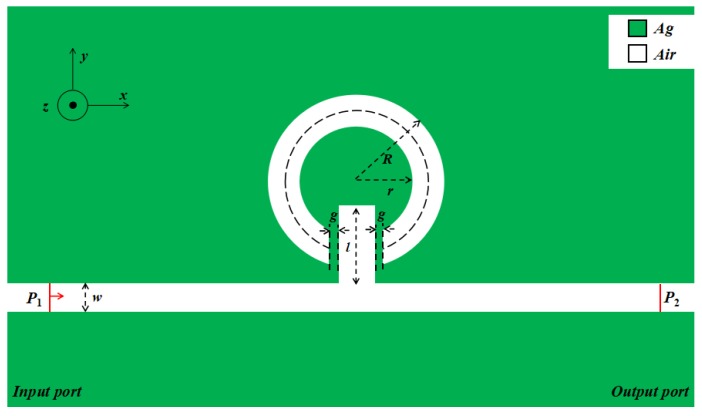
Two-dimensional schematic of the metal–insulator–metal (MIM) waveguide coupled with the notched ring resonator and stub.

Figure 1 shows a schematic of the MIM waveguide coupled with the stub and the designed notched ring resonator. The vertical symmetry axes of the two resonators and MIM waveguide coincide with each other. The width *w* of the waveguide is 50 nm to ensure that the waveguide only possesses a transverse magnetic field (TM_0_ mode) [31]. *g* represents both sides of the coupling distance between the stub and notched ring resonator. The inner diameter and external diameter of the notched ring resonator are *r* and *R*, respectively, while *l* represents the length of the stub.

The white part in Figure 1 represents the MIM waveguide and resonators, the filling medium is air (dielectric constant = 1), the green part represents metallic silver, and its dielectric constant is related to the frequency of incident light. Based on the Debye–Drude dispersion model [32], the relative permittivity of silver can be defined as

(1) $$\varepsilon \left(\omega \right)=\varepsilon_{\infty }+\frac{\varepsilon_{s}-\varepsilon_{\infty }}{1+i\omega \tau }+\frac{\sigma }{i\omega \varepsilon_{0}}$$

where $\varepsilon_{\infty }$ = 3.8344, τ = 7.35 × 10^−15^ s, $\varepsilon_{s}$ = −9530.5, and σ = 1.1486 × 10^7^ S/m are the infinite frequency permittivity, relaxation time, static permittivity, and conductivity of Ag, respectively. Although the dielectric parameters of silver were validated within 400–1200 nm, it is feasible to evaluate the Fano resonance of the MIM structure in this simulation experiment. COMSOL Multiphysics software based on the finite element method can be applied to solve the partial differential equation, and the transmission spectra of the coupling structure under different incident light frequencies can be obtained. The transmittance is defined as *T* = (*S*_21_)^2^, where *S*_21_ is the transmission coefficient from input to output (*P*_1_ to *P*_2_) [28].

## 3. Results and Discussion

**Figure 2:**
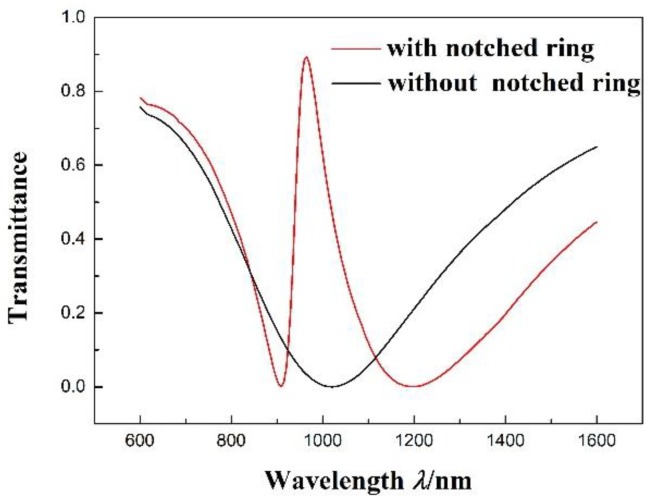
Transmission spectrum of the MIM waveguide with the notched ring resonator and without the notched ring resonator.

In Figure 2, the transmission spectra of the MIM waveguide with the notched ring resonator and the MIM waveguide without the notched ring resonator are shown as a red line and a black line, respectively. The parameters of the structure with *R* = 140 nm, *r* = 90 nm, *l* = 160 nm, *n* = 1 RIU, and *g* = 10 nm are presented. In the spectrum of the MIM waveguide without the notched ring resonator, the transmittance is about 0 at $\lambda_{\mathrm{dip}}$ = 1020 nm. However, in the spectrum of the MIM waveguide with the notched ring resonator, a narrow asymmetric resonance line has been found in the wider zone of the stopband; the transmittance is about 0 at $\lambda_{\mathrm{dip}}$ = 910 nm and about 0.9 at $\lambda_{\mathrm{peak}}$ = 965 nm, which is a typical Fano resonance [33] line-shape with a minimum and maximum. Therefore, the structure not only realizes the electromagnetically-induced transparency, but also realizes the Fano resonance.

**Figure 3:**
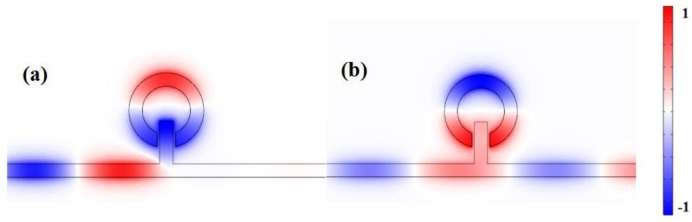
Contour profiles of the normalized *H_z_* field of different structures at (**a**) $\lambda_{\mathrm{dip}}$ = 910 nm and (**b**) $\lambda_{\mathrm{peak}}$ = 965 nm.

In order to better understand the internal mechanism of the change in the transmission spectra, the magnetic field distribution of the spectra at the resonance dip and peak is studied. Figure 3a,b show the fields *H_z_* of the plasmonic waveguide-coupled system at $\lambda_{\mathrm{dip}}$ = 910 nm and $\lambda_{\mathrm{peak}}$ = 965 nm. In Figure 3a, a weak coupling at the right side of the MIM waveguide is shown and has no SPPs coupled to it. There is a clear in-phase relationship between the lower part of the notched ring resonator and stub, and the relationship between the higher part and lower part of the notched ring resonator is anti-phase in Figure 3b.

**Figure 4:**
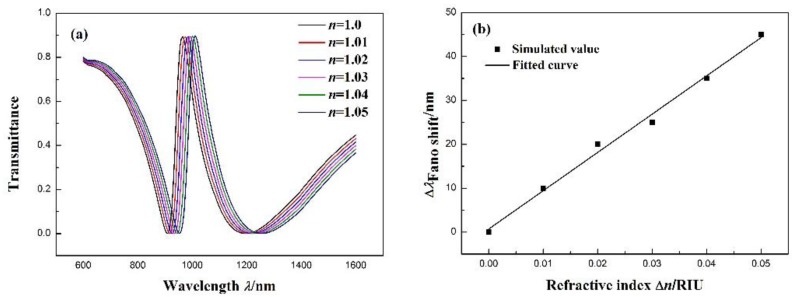
(**a**) Transmission spectra of the MIM waveguide coupled with the notched ring resonator and stub for different *n*. (**b**) Fitting line of the Fano resonance peak shift (Δλ) with the change in the refractive index (Δn).

Based on the propagation characteristics of the structure, the structure is applied to the refractive index sensor. The shift in the transmission spectra is due to the change in the refractive index. Therefore, the shift can be defined as the sensitivity of the sensor. The effect caused by the refractive index on the structure is studied by filling it with media of different refractive indices. Figure 4a presents the transmission spectra for different refractive indices from 1.00 to 1.05 RIU (interval of 0.01). As n increases, the transmission spectra red-shift an equal distance. Furthermore, Figure 4b indicates that there is a linear relationship between the wavelength shift in the Fano resonance peak and refractive index change Δn. With the increase in n to 1.05, the Fano resonance peak shifts to 1010 nm. The sensitivity (Δλ/Δn) can be obtained by linear fitting. The maximum sensitivity can reach 871.4 nm/RIU. In addition, the figure-of-merit (FOM) is an important parameter for the refractive index nanosensor. It is defined as (Δλ/Δn)/FWHM [34] and is 10.89 for the nanosensor.

**Figure 5:**
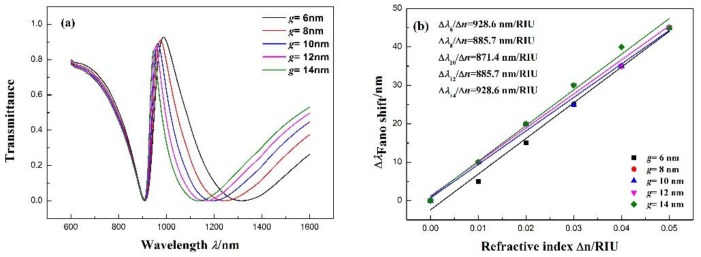

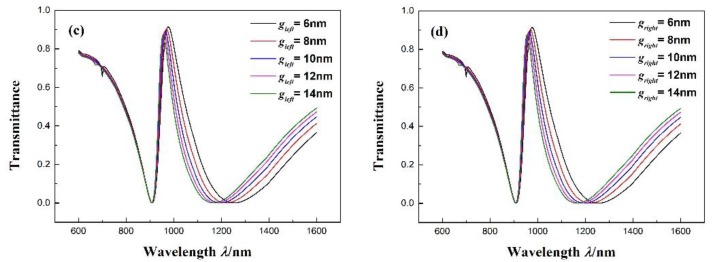
(**a**) Transmission spectra for different coupling distances *g* between the notched ring resonator and MIM waveguide. (**b**) Fitting line of the Fano resonance peak shift (Δλ) with the change in the refractive index (Δn). (**c**) Transmission spectra for different coupling distances *g_left_* between the notched ring resonator and MIM waveguide. (**d**) Transmission spectra for different coupling distances *g_right_* between the notched resonator and MIM waveguide.

Studying the effects of more than one coupling distance between the notched ring resonator and stub on the Fano resonance of the structure, we find that the *g* factor increases from 6 to 14 nm at intervals of 2 nm while maintaining the other parameters at *r* = 90 nm, *R* = 140 nm, *l* = 160 nm, and n = 1 RIU. The transmittance of the Fano resonance peak increases slightly, and the position of the dip remains unchanged. In addition, the Fano resonance peak red-shifts slightly in Figure 5a. Figure 5b shows the Fano resonance peak shift with the change in Δn. With the increase in n, the transmission spectra red-shift an equal distance. The fitting calculation indicates that the maximum sensitivity can reach 928.6 nm/RIU with *g* = 6 and 12 nm, with FOMs of 6.40 and 14.29, respectively. Besides, in order to understand the effects of the symmetry breaking. We change the *g_left_* factor increases from 6 to 14 nm at intervals of 2 nm while keeping the *g_right_* = 10 nm as well to the right side. The transmittance spectra are as shown in Figure 5c,d. Furthermore, the effects on the Fano resonance of the asymmetric structure are similar to the symmetric structure.

**Figure 6:**
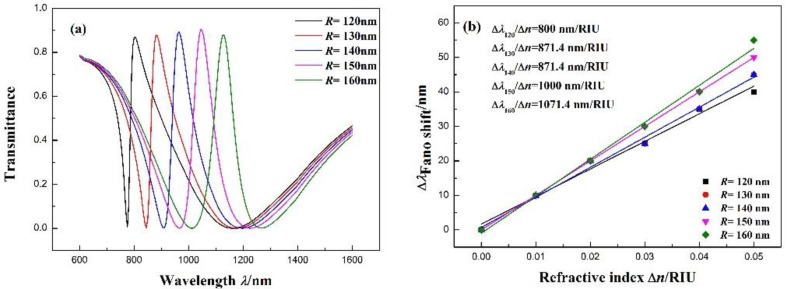
(**a**) Transmission spectra for different external diameters of the notched ring resonator *R*. (**b**) Fitting line of the Fano resonance peak shift (Δλ) with the change in refractive index (Δn).

We also study the impact of different external diameters on the transmission spectra. The factor *R* increases from 120 to 160 nm at intervals while maintaining the other parameters at *l* = 160 nm, n = 1.00 RIU, and *g* = 10 nm, and the width of the notched ring is 50 nm. As shown in Figure 6a, with the increase in the equal distance of *R*, the Fano resonance red-shifts an equal distance. The Fano resonance is attributed to the coupling between the broadband spectrum and narrowband spectrum. The broadband spectrum signal is generated by the stub, while the narrowband spectrum signal is generated by the notched ring resonator. The increase in *R* results in the growth of the narrowband spectrum resonance wavelength, which leads the Fano resonance to red-shift and changes the Fano spectral asymmetry. The fitting line of the Fano resonance peak shifts with the refractive index change (Δn) in Figure 6b. The fitting outcome indicates that the maximum sensitivity can achieve 1071.4 nm/RIU with *R* = 160 nm, and its FOM is 14.29. As can be seen from the sensitivity in Figure 6b, the spectral asymmetry is strongly related to the sensitivity of the sensor.

**Figure 7:**
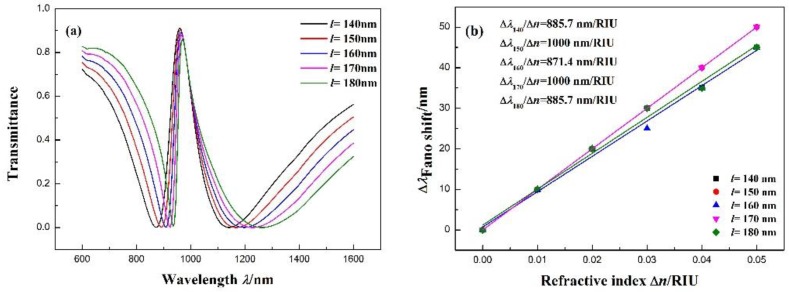
(**a**) Transmission spectra for different stub lengths *l*. (**b**) Fitting line of the Fano resonance peak shift (Δλ) with the change in refractive index (Δn).

Moreover, we investigated the influence of different stub lengths *l* on the transmission spectra. *l* increases from 140 to 180 nm (interval 10 nm) while maintaining the other parameters at *R* = 140 nm, *r* = 90 nm, n = 1.00 RIU, and *g* = 10 nm. As shown in Figure 7a, with the increase in the equal distance of *l*, the transmission spectra red-shift slightly. Figure 7b describes the relationship of the Fano resonance peak shift with the refractive index Δn. The fitting calculation shows that the maximum sensitivity can achieve 1000 nm/RIU with *l* = 150 and 170 nm, with FOMs of 11.11 and 12.5, respectively.

## 4. Conclusions

We used the finite element method to study the transmission characteristics of a MIM waveguide coupled with a stub and notched ring resonator. The results indicate that the coupled structure clearly generates typical Fano resonance. Its propagation characteristics indicate that the Fano resonance in the transmission spectra depends on the geometric parameters of the notched ring resonator, and they are insensitive to small changes in the length of the stub and the coupling distance between two resonators. This characteristic significantly reduces the difficulties faced when using micro-/nano-processing technology. In addition, the Fano resonance produced by the structure is extremely sensitive to the change in different surrounding media, and the sensitivity reaches 1071.4 nm/RIU after optimizing the structural parameters, which provides a new method based on the MIM waveguide for detecting changes in the refractive index.
